# Current Status of Therapeutic Approaches against Peripheral Nerve Injuries: A Detailed Story from Injury to Recovery

**DOI:** 10.7150/ijbs.35653

**Published:** 2020-01-01

**Authors:** Ghulam Hussain, Jing Wang, Azhar Rasul, Haseeb Anwar, Muhammad Qasim, Shamaila Zafar, Nimra Aziz, Aroona Razzaq, Rashad Hussain, Jose-Luis Gonzalez de Aguilar, Tao Sun

**Affiliations:** 1 Neurochemicalbiology and Genetics Laboratory (NGL), Department of Physiology, Faculty of Life Sciences, Government College University, Faisalabad, 38000 Pakistan.; 2 Center for Precision Medicine, School of Medicine and School of Biomedical Sciences, Huaqiao University, Xiamen, Fujian Province, 361021 China; 3 Department of Zoology, Faculty of Life Sciences, Government College University, Faisalabad, 38000 Pakistan; 4 Department of Bioinformatics and Biotechnology, Government College University, Faisalabad, 38000 Pakistan; 5 Department of Neurosurgery, Center for Translational Neuromedicine (SMD), School of Medicine and Dentistry, University of Rochester Medical Center, 601 Elmwood Ave, Box 645, Rochester, NY 14642, USA; 6 Université de Strasbourg, UMR_S 1118, Strasbourg, France; 7 INSERM, U1118, Mécanismes Centraux et Péripheriques de la Neurodégénérescence, Strasbourg, France

**Keywords:** Peripheral Nerve Injury, Pathophysiology, Surgical interventions, Non-surgical intervention, Plant-derived compounds

## Abstract

Peripheral nerve injury is a complex condition with a variety of signs and symptoms such as numbness, tingling, jabbing, throbbing, burning or sharp pain. Peripheral nerves are fragile in nature and can easily get damaged due to acute compression or trauma which may lead to the sensory and motor functions deficits and even lifelong disability. After lesion, the neuronal cell body becomes disconnected from the axon's distal portion to the injury site leading to the axonal degeneration and dismantlement of neuromuscular junctions of targeted muscles. In spite of extensive research on this aspect, complete functional recovery still remains a challenge to be resolved. This review highlights detailed pathophysiological events after an injury to a peripheral nerve and the associated factors that can either hinder or promote the regenerative machinery. In addition, it throws light on the available therapeutic strategies including supporting therapies, surgical and non-surgical interventions to ameliorate the axonal regeneration, neuronal survival, and reinnervation of peripheral targets. Despite the availability of various treatment options, we are still lacking the optimal treatments for a perfect and complete functional regain. The need for the present age is to discover or design such potent compounds that would be able to execute the complete functional retrieval. In this regard, plant-derived compounds are getting more attention and several recent reports validate their remedial effects. A plethora of plants and plant-derived phytochemicals have been suggested with curative effects against a number of diseases in general and neuronal injury in particular. They can be a ray of hope for the suffering individuals.

## Introduction

The nervous system is a complex network of nerves which coordinates its activities by the transmission of signals to and from different body regions. It senses the changes in the environment which influence the body 1 and is classified into two portions, the central nervous system (CNS) and peripheral nervous system (PNS). The CNS is comprised of spinal cord and brain, whereas nerves make the PNS which are the constrained bundles of prolonged fibers or axons and functionally integrate different parts of the body with CNS 2. The PNS includes several types of nerve fibers such as spinal and cranial nerve fibers which build up a communication pathway between CNS and peripheral areas of the body. Sensory signals of our body are conveyed to the CNS with the help of sensory nerve fibers of PNS whereas; the generated response is delivered by motor nerve fibers of PNS to the target end 3. Thus, for physiological regulation of the entire living system, the continuity of this communication is pivotal.

Peripheral nerve fibers, the most delicate and fragile structure of our body, are prone to get damaged easily by crush, compression, or trauma. Their damage manifests as abnormalities in the brain's communication with the target organs and muscles 4. Peripheral nerve injuries (PNIs) fall amongst the most pivotal issue regarding the health status because of their higher prevalence. These injuries affect motor activity and also cause the loss of sensation in the respective part of the body 5. Thus the PNIs adversely affect the brain's functions and communication with the target organs or muscles. These injuries affect behavior, mobility, perception, consciousness, sensations of skin and joints and most often result in a life-long disability for the affected individual 6,7. These injuries are difficult to treat because of various underlying factors like location, intensity and type of nerve injury 8. Although, in this aspect, different strategies and medicinal interventions have been acquired and practiced. The future of PNI treatment depends on exploiting a recovery of sensory and motor function after injury to the nerve. Approach for the sustenance of neuromuscular junctions is significant for allowing re-innervation of muscles after persistent denervation of muscles and reducing injury to cell body as well 9.

The present review will strive to delineate the series of pathophysiological changes at the site of nerve injury in a comprehensive and coherent manner. This can lead to the adoption of innovative approaches with regard to the treatment of PNIs. The aim of this review is to enhance the understanding of PNIs, consequences, and pathophysiology of PNIs. At present, the available surgical and non-surgical remedies for PNIs have been brought to light. The literature was searched via several e-sites, including PubMed, Springer Link, Science Direct Scopus, Elsevier, and some other pertinent medical journals, highlighting the informs in this field for surgeons and clinical practitioners. Keywords used for the literature search are “Peripheral Nerve Injuries”, “Consequences and pathophysiology”, “Surgical Remedies”, and “Non-Surgical Remedies”.

## Classification of peripheral nerve injuries and their consequences

Peripheral nerve injuries (PNIs) have been classified by scientists into different grades depending upon the severity. This classification scheme helps scientists and physicians to discuss effectively the nerve pathophysiology and to determine the appropriate treatment. The PNIs were classified by Sir Sydney Sunderland and Sir Herbert Seddon. Seddon classified the PNIs into 3 grades on the basis of the presence of demyelination and the extent of damage to the axons and the connective tissue of the nerve. Sunderland then gave further subdivision on the basis of discontinuity of several layers of connective tissues in peripheral nerve 27.

### 1. Seddon's classification of nerve injuries

In 1943, the injuries of peripheral nerves were classified into three main grades by Seddon, termed as Seddon's classification. These include neuropraxia, axonotmesis and neurotmesis 3. The brief description of these injuries with their consequences is given in table 1.

### 2. Sunderland's classification of nerve injuries

In 1951, Sunderland expanded this scheme of classification to five grades to distinguish the extent of damage in connective tissues. As it is explained that neuropraxia is the condition which involves the slight crush of nerve fiber or compression which also harms the myelin sheath, leads to the blockage of impulse conduction. The other grade of injury is axonotmesis that was first introduced by Seddon and it was further divided into three grades by Sunderland. The grade 2 damage (Sunderland division) indicates the damage in which axon and the myelin sheath become disconnected but connective tissues' continuity remain conserved 20. Thus, this leads to the denervation of targeted areas and causes the disturbance of sensory/motor function. It may take several weeks to several months for complete functional retrieval subsequently the axonal regeneration is essential, but this type of injury does not require any surgical intermediation 27. In grade 3 damage, the axon and axonal sheath become disconnected along-with the endoneurial layer, whereas the layers of connective tissues remain intact and the functional retrieval is more difficult. In grade 4 injury, there is only continuity of epineurium whereas all the other layers and axonal sheath become disconnected. Thus the grade four damage (Sunderland division) is much more severe 21.

The grade 5 Sunderland classification of injury is termed as neurotmesis (Sometimes grade 4 can also be termed as neurotmesis), indicates that all the three layers (endoneurium, perineurium, epineurium) and axonal myelin sheath become disconnected 39. These types of whole nerve laceration/transection injuries require mandatory and prompt surgical intermediation for the achievement of complete functional retrieval 19. In few situations, the term grade 6 injuries might be used and entitles the injury with the mixed type of injury such as due to a gunshot, stabbed wound, or closed traction instigating partial nerves injuries called “a neuroma-in-continuity”. This represents a mixture of any of the previously described five grades of injury and is most challenging for surgeons to tackle 23. The summary of Seddon and Sunderland classification is described in table 2.

## Pathophysiology of peripheral nerve injury

The peripheral nerve injury elicits a cascade of changes in physiological as well as the metabolic level at the injured site and several changes also happen in the soma of injured neuron 24. A neuron is divided into two segments as distal and proximal to the site of injury and both are significantly different from each other 48. The distal part suffers the Wallerian degeneration (WD) while the proximal part goes through the retrograde degenerative changes as well as instigates the process of regeneration. The process of WD initiates within 24-48 hours following injury and emerges at the distal end of the abrasion in case of severe nerve injury 26,27. It was first described by Waller in 1850. It involves invasion by myelomonocytic cells that destroy myelin and initiate mitosis in Schwann cells 28. When there is continuous disconnection of axons, a sequence of alterations occurs at distal and proximal sites of injury. The ends of the discontinued axons stamp themselves and become swollen within a few hours of injury. The site of individual axon becomes degenerated which is proximal to the subsequent node of Ranvier. Moreover, the disintegration of neurofilaments and cytoskeleton also takes place 29-31.

### 1. Degenerative changes at the distal end

On the injured site, the distal part of axon swells and Schwann cells allow the calcium influx which triggers the proteases discharge. This event causes a decrease in impulse transmission and leads to the breakdown of myelin 32. The activation of proteases leads to the degradation of neurofilaments, mitochondria, endoplasmic reticulum, and cytoskeleton of the axon 33. The shrinkage of nerve's skeleton happens at the end of the Wallerian degeneration with retraction of axon terminals from the target. Initiation of the inflammatory process and edema formation occurs when there is a more severe type of injury. Dense scar of the fibrous tissues is formed as a result of fibroblasts proliferation at the injury site which further flares up the inflammation at the injured portion 57. Moreover, the myelin sheath shows beaded appearance and fatty enlargement and suffers the loss of myelin and Schwann cells integrity which allow the disintegration of axonal membrane and formation of myelin fragments. This phenomenon collectively slackens the initiation of axonal regeneration reaching proximal stump 58.

### 2. Degenerative and regenerative changes at the proximal end

**Figure 1:**
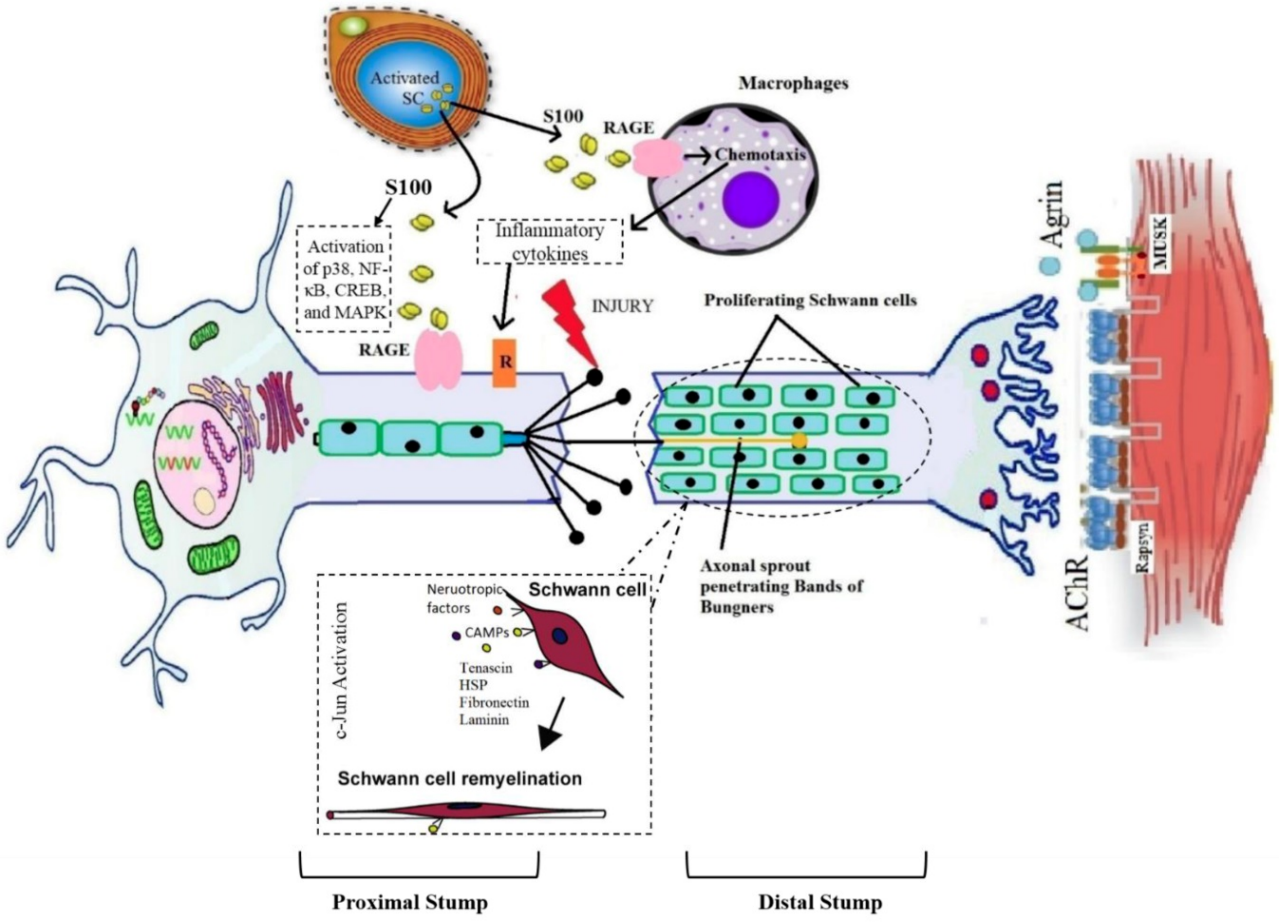
Graphical representation of pathophysiology of wallerian degeneration

The proximal part of the injured neuron suffers from retrograde degenerative changes before the initiation of the regenerative process. The cell body swells up and becomes rounded; the Nissl's granules become degenerated and weakly stained. Furthermore, the nucleus and endoplasmic reticulum (ER) are shifted eccentrically. This phenomenon is termed as chromatolysis 36. The process of regeneration initiates with the reversal of all retrograde degenerative changes. As the Schwann cells stop making myelin, they cause macrophage activation, which leads to the phagocytosis of myelin sheath deposits 37. In addition to clearing myelin debris, macrophages and Schwann cells also produce cytokines i.e., interleukin-6 to promote axonal growth 38. As debris clears, the regeneration starts at the proximal end of the injured site and continues toward the distal end. The Schwann cells play a role in guiding the cytoplasmic extensions of the axonal sprout between the basement membranes of two nerve ends 39. Bungner's bands are formed by the alignment of Schwann cells in longitudinal columns along with cleaned endoneurial tube which guides the sprouting axons towards the targeted tissue for re-innervation 40. These bands then release the growth factors including fibroblast growth factor (FGF), nerve growth factor (NGF), interleukin-like growth factor (IGF), ciliary neurotrophic factor (CNTF), brain-derived neurotrophic factor (BDNF), and vascular endothelial growth factor (VEGF) 64,65. The fibronectin and laminin found inside the basal lamina Schwann cells and the released neurotrophic factors monitor the sprouting axon into endoneurial column 43,44. The tip of every axonal sprout has specified growth cones that comprised of numerous filopodia which adhere themselves to the basal lamina of Schwann cell 45. The pathophysiology of Wallerian degeneration is elaborated in figure 1.

The process of chemotactic and communication repulsion and attraction regulates the fortune of an axon that needs to be regenerated. The frequency of regeneration of axon is estimated by alterations within the soma, growth cone stability at the axonal sprout tip, and the hindrance of damaged tissue between end organ and soma 69. In humans, axonal regeneration occurs at a rate of almost 1 mm/day. Thus moderate to severe type of injuries take months or even years to heal 47. The PNI lead to the extensive changes in the neuronal expression of thousands of the genes which includes numerous transcription factors 40. The PNS has the capability to self-regenerate and a large number of factors involved in the peripheral nerve regeneration are highlighted in table 3.

## Why we need to go for treatment

Even after a long healing period full functional re-innervation without any complication is not possible. In the case of grade III injury 16, retraction of the severed nerve fiber ends happens due to elastic endoneurium which causes local trauma. It leads to a significant inflammatory response. Fibroblast proliferation aggravates the process and a dense inter-fascicular scar is formed. This kind of injury distracts the axonal regeneration and endoneurial tubes remain denervated. If the endoneurial tube does not receive a regenerating axon, the progressive fibrosis ultimately demolishes it. In the IV and V grade injuries, activated Schwann cells and fibroblasts cause vigorous cellular proliferation 64. Local vascular trauma leads to macrophage accumulation. In these types of injuries, the nerve ends become an irregular mass of Schwann cells, fibroblasts, macrophages and collagen fibers. Regenerating axons face such disorganized proximal stump and come across a rough barrier that delays further growth. In case of disconnection of cell bodies from axons, a programmed cell death pathway activates within 6 hours of the injury and is called chromatolysis 65. The process of regeneration is limited or complicated in case of severe injuries and results in the aggravation of muscular atrophy and the patient then need to go for surgical interventions to attain recovery.

## Available treatment approaches/strategies

**Figure 2:**
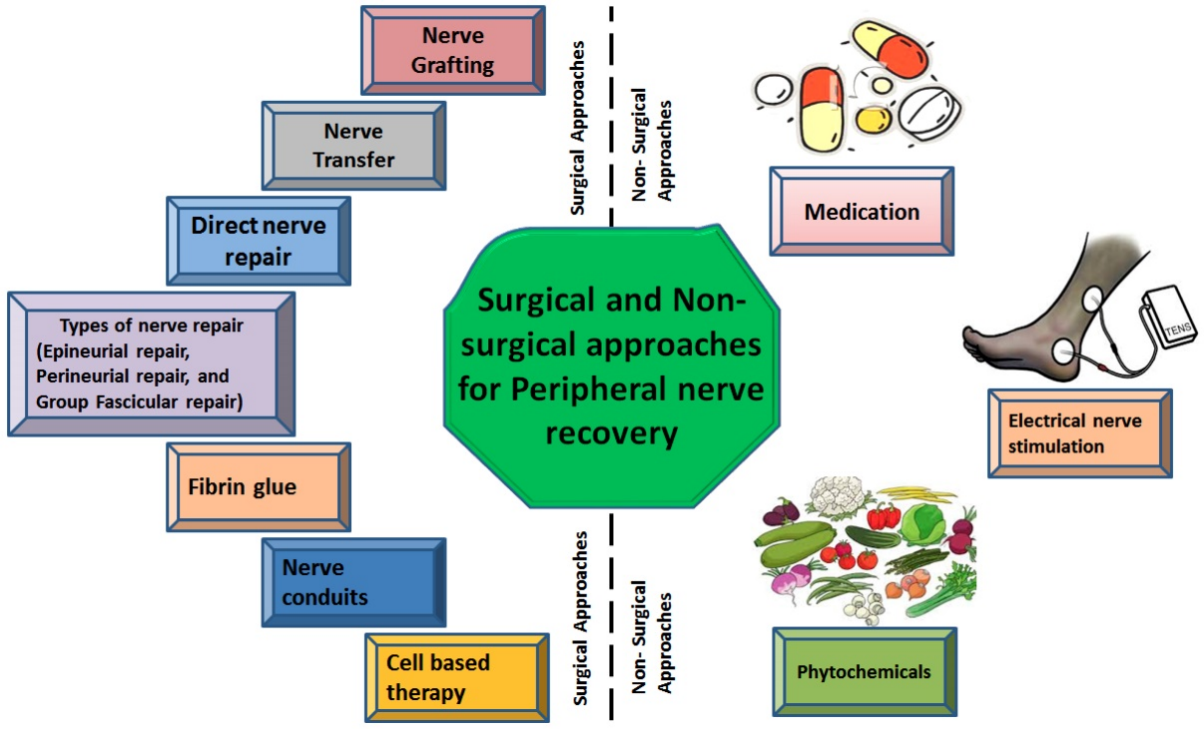
Types of surgical and non-surgical intrusions against peripheral nerve injury

The recovery time of the injured nerve depends on various external factors including early nerve exploration and nerve repair. However, it should be known that axonal regeneration rate is as slow as 1-2 mm/day and there is no treatment that can accelerate this process 66. The irreversible motor unit degeneration starts 12-18 months after denervation of the muscle but may persist for 26 months 67. Recovery of sensory regeneration may take longer. Additional injury at target muscles or an injury in peripheral supportive tissue delays the recovery more than usual. The features of the injury define the type or timing of the surgical nerve repair. There are three types of wound including tidy, untidy and closed traction injuries 68. The tidy wound may be made by a glass or a scalpel, which has sharp edges and primary repair is a preferable treatment option. Untidy wound samples are open fractures or gunshot wounds with extensive tissue damage and infection and cannot be repaired immediately. Closed traction injuries have retracted and damaged nerves, vessels and peripheral supportive tissues. Closed traction injuries have the worst outcomes of all wounds 69. Furthermore, in the next section possible surgical and non-surgical approaches against PNIs have been discussed and are also illustrated in figure 2.

### 1. Surgical therapeutic approaches for peripheral nerve recovery

There are six most common types of therapeutic techniques used for sensory and motor functional recovery following a traumatic injury particularly PNIs.

#### 1.1 Direct nerve repair

A gold-standard method for treatment of axonotmesis and neurotmesis is the direct nerve repair with microsurgical techniques to provide endurance or continuity between the distal and proximal part of the nerves 70. When there is need of surgical repair for transected nerves or nerve damage demanding deletion, the best outcomes are attained with a direct nerve repair technique 71. There are 3 categories of nerve repair techniques, including epineurial repair, perineurial repair, and group fascicular repair. For comprehensive detail, please consult this article 72.

##### 1.1.1 Epineurial repair

This technique of nerve repair is used to suture the lacerated nerves and is applicable for both neural repairs (primary and secondary) and also involves the sutures only on the outer sheath of the nerve 73. It has advantages like minimum magnification, short execution time, not assaulting the intra-neural contents, and technical ease 74. Following the nerve repair, it is the most important method to achieve the tension-free natural connection with no loss of the nerve tissue and precise alignment of the nerve fascicles 103.

##### 1.1.2 Perineurial repair

Initially, this technique was described by Hashimoto and Langley in 1917 104. It is a better choice for major acute nerve lacerations and for suturing the epineurium because of simple and faster method and also involves the minor disruption of the internal structure of a nerve. While discussing the neurophysiological and morphological aspects of this nerve repair technique, it has proved to be more valuable in terms of soothing and neuronal pathways after good localization of fibers at nerve terminals 105. Some of the drawbacks of this technique include greater fibrosis at the nerve suture site, extended operative period, fasciculi discontinuity on a one-to-one basis 75,78.

##### 1.1.3 Group fascicular repair

It is an easy technique when the nerve is lacerated and the branches in transected nerves are well organized and identified in the main trunk 79. The sensory and motor fascicles can be coordinated correctly as well as the cross-innervation of motor sensory nerves can be evaded. Nevertheless, it is not very practical at the present time because of many disadvantages like long operative procedure (Riley and Lang, 1984).

## Disadvantages of nerve repair

The major drawback of nerve repair is that it does not assure the functional recovery which may lead to the partially reversible neuronal atrophy and fall the production of neurotrophic factors to hinder the accelerated regeneration 81. One of the major factors affecting the nerve repair is that it involves the accurate connection of two sides of the transacted nerve with very few sutures while dissecting the nerve endings to the extent necessary for appropriate alignment with slight tension 79. In case, if only sensory and motor parts of the nerve are precisely connected, better functional recovery could be achieved. So, misalignment of sensory and motor axons can deteriorate the recovery process leading to a long time in the activity of targeted muscles which can undergo denervation tempted atrophy.

### 1.2 Nerve grafting

Nerve grafting is a technique used to bridge the nerve gaps larger than 2 cm by transplanting the nerve donating from the same species. In this method, the gap should cut longer than the lesion; the connective tissue of the fascicles should be dismembered rather than every single fascicle. The fascicles dissection should be at the proximal and distal ends in relation to the lesion within normal tissue 79. Several factors should be considered for selecting the donor for nerve including the diameter of host and donor nerves, length of nerve grafts, number of fascicles, fascicular pattern, cross-sectional area and shape, and patient preferences 71. There are two types of nerve grafting including nerve autografts (autologous nerve grafts) and nerve allografts.

#### Nerve autografts

Autografts (autologous) are the gold-standard option for peripheral nerve repair 82. As reported in the literature, autologous nerve grafting has better recovery for long nerve deficits (>3 cm), more proximal injuries and critical nerve injuries 83. Generally, donor nerve grafts are extracted from expandable sensory nerves such as lateral and medial antebrachial nerves, ulnar nerve's branch (dorsal cutaneous), radial nerve's superficial sensory branch, and lateral femoral cutaneous nerve 81. Depending on the severity of the injury, different nerve autografts have been used which include cable, single, vascularized, interfascicular and single nerve autografts 84.

**Advantages:** Autologous nerve grafting has best results due to involvement nerve regeneration promoting factors, including Schwann cells, basal lamina, neurotrophic factors and adhesion molecules as well as having non-immunogenic effects 85.

**Disadvantages:** Despite the beneficial results, the autologous nerve grafts has some limitations including limited tissue availability, the graft, donor-site morbidity, loss of nerve function, scarring, second incision, neuroma formation, limited supply, and potential difference in tissue size 85.

#### Nerve allografts

Nerve allograft is one of the most favorable alternatives to nerve autografts. The allograft nerves are collected from a cadaver or donor for nerve grafting 86. The availability of cadaveric nerve allografts are highly abundant and contain both endoneurial microstructure and Schwann cells (SC) of the donor which support the regeneration process 87. The systemic immunosuppression is essential in this technique to avoid graft rejection and the donor's SCs have a dual role; act as both remyelination assistance and facultative antigen. Moreover, the systemic immunosuppression is temporary and can be removed once the occurrence migration of host SCs is adequate (approximately 24 months).

**Advantages:** It is readily accessible, circumvents morbidity of the donor site and availability of unlimited supply.

**Disadvantages:** The recovery results are good, but the process is too expensive and requires experience to perform 88,89. Immunosuppression has many side effects including opportunistic infections and tumor formation.

Currently, scientists are focusing on acellular human nerve allografts with the aim to eliminate the need for immunosuppressants 90. The decellularization process is performed using chemical detergents, enzyme degradation or irradiation 91. Acellular nerve allografts are removed from Schwann cells and myelin, but the internal neuronal structure and extracellular matrix (collagen, laminin and growth factors) are preserved 92. Regeneration process with acellular allografts involves host's migrated Schwann cells. Thus, even acellular nerve grafts show good outcomes in trials, they are still inefficient in long nerve repairs. In the future, acellular grafts supplemented with seed cells and growth factors may improve the surgical repair outcomes of a large gap of PNIs 93. Hence, in spite of multifarious use and improvements in grafts, there is still a need for further improvements with better prognosis. If the issue of immunosuppression can be resolved, it will be a great achievement in this field.

### 1.3 Nerve transfer

It is a surgical method which is used for the treatment the nerve injury after the complete loss of sensory and muscle functions 94. In case of severe proximal nerves injury, it might be a sole reconstructive choice in hand. The reconstruction is preferred with the distal motor nerve transfer through the use of extended nerve grafts for the middle and high-level injuries. This method allows the segmentation in the un-injured and un-scarred tissues planes and lessens the regeneration distance and time 95. Moreover, in this method, auxiliary motor units are surgically re-established and re-organized to retrieve the sensibility and functional loss 79.

**Advantages:** It can be considered superior to the nerve grafting as the surgical region during the nerve transfer is away from the injury site and use recognizable and healthy tissues rather than scarred or crushed tissues present at the injury site. It preserves the anatomy and biomechanics of the nerve and allows its reinnervation to the targeted muscle 96.

**Disadvantages:** It takes about several months for clinical results after the nerve transfer and it demands technical expertise. It is a quite expensive method and availability of donor limits its validation 97. By considering the drawbacks, nerve transfer cannot be taken as a standard treatment method. Although it encompasses a lot of benefits, unfortunately, its adverse effects cannot be neglected. For more comprehensive detail, please consult this article 98.

### 1.4 Fibrin glue

Fibrin glue enables primary sutureless repair by using an adhesive material known as fibrin sealants. It is considered as an efficient technique to avoid suturing for nerve cooptation.

**Advantages:** Repair with fibrin glue ensures a shorter recovery time, less fibrosis and decreased inflammatory reactions 99. The most important advantage of the fibrin glue is its quick and easy application in emergency conditions whenever there is the absence of experienced surgeon for nerve repair but it's not applicable for severe injuries 100. An ideal sealant should have specific mechanical, structural and biological properties as well as it must not hinder the regeneration process.

**Disadvantages:** The biggest disadvantage of commercially available sealants is the use of human blood that can result in the transmission of infection, fibrosis, toxicity and necrosis 101.

Taking this into account, a new snake venom-derived heterologous fibrin sealant (HFS) has been discovered. It can prevent the loss of fluid, decreases the time of surgery and reduces hemorrhage 102. In the future, if more affordable and authentic nerve sealants are discovered, it will be a great breakthrough in this field. Moreover, this effort may reduce the number of individuals suffering from PNI. For more detail, please consult this article 103.

### 1.5 Nerve conduits

Nerve conduits serve as a bridge between the proximal and distal stumps of the injured nerve. They provide a scaffold for axonal regeneration and can be used as an alternative to the nerve autograft. In recent years, scientists have focused on the development of conduits as an alternative treatment, especially for complex defects. 104. In this technique, distal and proximal stumps are inserted into 2 endings of the nerve conduit, allowing the axonal regeneration from proximal stump via the conduit and discriminatingly grow into their usual pathways in distal nerve stump. The conduits prevent the incursion of nearby tissues into a slit between the stumps. On top of that, these conduits are rich in neurotrophic factors that enhance the regeneration of axon following a nerve injury 105. The most important advantage of a conduit is its ability to provide an ideal microenvironment for neuronal recovery. For this purpose, an ideal nerve conduit should have properties like porous, flexible, thin, biocompatibility, permeability, flexibility, biodegradability, compliance, neuroinductivity and neuroconductivity with an appropriate surface 106,107. Conduits are categorized into two groups according to their materials as synthetic conduits and biological conduits.

### Synthetic nerve conduits

They are further categorized into degradable and non-degradable conduits. **Non-degradable nerve conduit** materials include silicone, elastomeric hydrogel and porous stainless steel. Although the reconstruction with these materials is successful, the possibility of foreign body reaction, scar formation, inflammation of neighboring tissues, lack of stability and the inflexible structure have curbed their extensive use. Another drawback is the requirement of a second surgery for conduit removal. Commonly used **degradable nerve conduit** materials include collagen, polyesters (e.g., polyglycolicacid (PGA)), chitosan, polylactic acid (PLA) and hydrogel. These materials induce only minimal foreign body reaction and effective nerve regeneration with these conduits has been reported 108,109. The most reliable nerve conduit is **collagen-based nerve conduits**. There are many Food and Drug Administration (FDA) approved collagen-based conduits such as NeuraGen, NeuroFlex, NeuroMatrix, NeuroWrap and NeuroMend. The collagen-based conduits are restorable and flexible. They are preferred as they cause minimal scar formation, allow nutrient transfer and provide a suitable environment for nerve regeneration without any compression neuropathy 110.

**Biological nerve conduits:** They include autologous arteries, veins, muscle, human amniotic membrane and umbilical cord vessels. Major advantages of biological conduits are non-activation of foreign body reaction, biocompatibility and enhanced migration of supportive cells. These biomaterials have been widely used for the repair of a short gap (<3 cm) nerve injuries and the outcomes are consistent with those of nerve grafts 111,112. As these type of conduits are only applicable in case of short gaps, the functional recovery for extensive damage with larger gaps is still questionable. Although, we have nerve conduits, the synthetic conduits, for larger gaps but their use has been banned due to a detrimental drawback. There is a need to explore authentic nerve conduits suitable to bridge up larger gaps with no menacing effects to completely replace the nerve autografts.

Currently, the choice of material for nerve conduits has shifted towards the more biocompatible synthetic polymers such as polyglycolic acid (PGA) and poly-lactidecaprolactone (PLCL). Neurotube is a PGA nerve conduit, while Neurolac is a PLCL conduit. Neurotube and Neurolac were designed to bridge the gaps between 8 mm to 3 cm and more than 3 cm respectively 106. Fibrin, gelatin, keratin and silk are other biopolymer conduit materials that are still under experimental evaluation 113,114. For more detailed information, please consult these articles 103,115. However, these polymers have insufficient biocompatibility resulting in cellular attachment, differentiation and proliferation. So, these factors should be taken into account before introducing them into clinical trials.

#### 1.6 Cell-based therapy

The basic limitations of present therapies are slow nerve regeneration and insufficient filling of large gaps. To overcome these limitations, cell-based therapy was designed to provide supportive cells to the lesion site with the aim to accelerate nerve regeneration which could replace the use of all other available surgical therapies 116. Most extensively studied therapeutic models are Schwann cells (SCs), but remarkable improvements were also achieved with different types of stem cells as well. Cell-based therapy is performed with stem cells owing to their self-renewal ability and capacity for differentiation into specialized cell type 117. The SCs, bone marrow-derived mesenchymal stem cells (BMSCs), adipose-derived mesenchymal stem cells (ADSCs) and pluripotent stem cells are the primary cell types which are used for cell-based therapy.

### Schwann cells-based therapy

Schwann cells (SCs) are the most significant and first-choice seed cells as they are the primary functional cells of the peripheral nervous system that promote myelination and regeneration 118. They play a crucial role in nerve regeneration by promoting the production of neurotrophic factors mainly nerve growth factor (NGF), brain-derived neurotrophic factor (BDNF), ciliary neurotrophic factor, platelet-derived growth factor and neuropeptide Y 119. In addition to that, SCs are capable of self-proliferation, immune system modulation, remyelination and migration. All these factors account to the amelioration of injured nerve regeneration and healing. In cell-based therapies, neural crest cells are the main source of SCs. The SCs seeds are transplanted in nerve conduits which accelerate the axonal regeneration. Unfortunately, they encompass slow expansion to large numbers and are hard to obtain 118.

### Other cell-based therapies

**Embryonic stem cells (ESCs)** have preferable advantages such as providing an unlimited source of cells, good differentiation potential and long-lasting proliferation capacity. However, ethical concerns are the major problem when these cells are used for transplantation. **Neural stem cells (NSCs)** can differentiate into neurons and glial cells, but their use is limited because these cells are difficult to harvest and there is a risk of neuroblastoma formation 120. **Bone marrow-derived stem cells (BMSCs)** have the potential to differentiate into SC-like cells (BMSC-SCs). However, studies have shown that the differentiation potential of BMSCs is not as strong as NSCs 118. **Fetal stem cells** can be derived from amniotic fluid, amniotic membrane, umbilical cord and Wharton's jelly. Both amniotic tissue-derived stem cells (ATDSCs) and umbilical cord-derived mesenchymal stem cells (UC-MSCs) have differentiation and proliferation potential. Major advantages of fetal-derived stem cells are their easy accessibility and less immunoreactivity. Unluckily, the ethical concerns are the main disadvantage of fetal-derived stem cells also. **Adipose stem cells (ADSCs)** also exhibit strong angiogenic potential and cause augmented neuronal injury perfusion. 121. **Skin-derived precursor stem cells (SKP-SCs)** are found in the dermis and can differentiate into any kinds of cells like neurons and glial cells. It is reported that SKP-SCs accelerate nerve regeneration. **Hair follicle stem cells (HFSCs)** have a unique feature of differentiation into SCs directly without any genetic intervention. Animal studies have been reported with improved nerve repair by using HFSCs. Several drawbacks associated with the stem cells have led to the use of alternative cells like induced pluripotential stem cells (iPSCs). The **iPSCs** show enhanced neuronal regeneration, but tumorigenicity, need for immunosuppression and chromosomal aberrations have limited their use 122. For more details on this aspect, please consult these worth reading articles 123,124.

**Figure 3:**
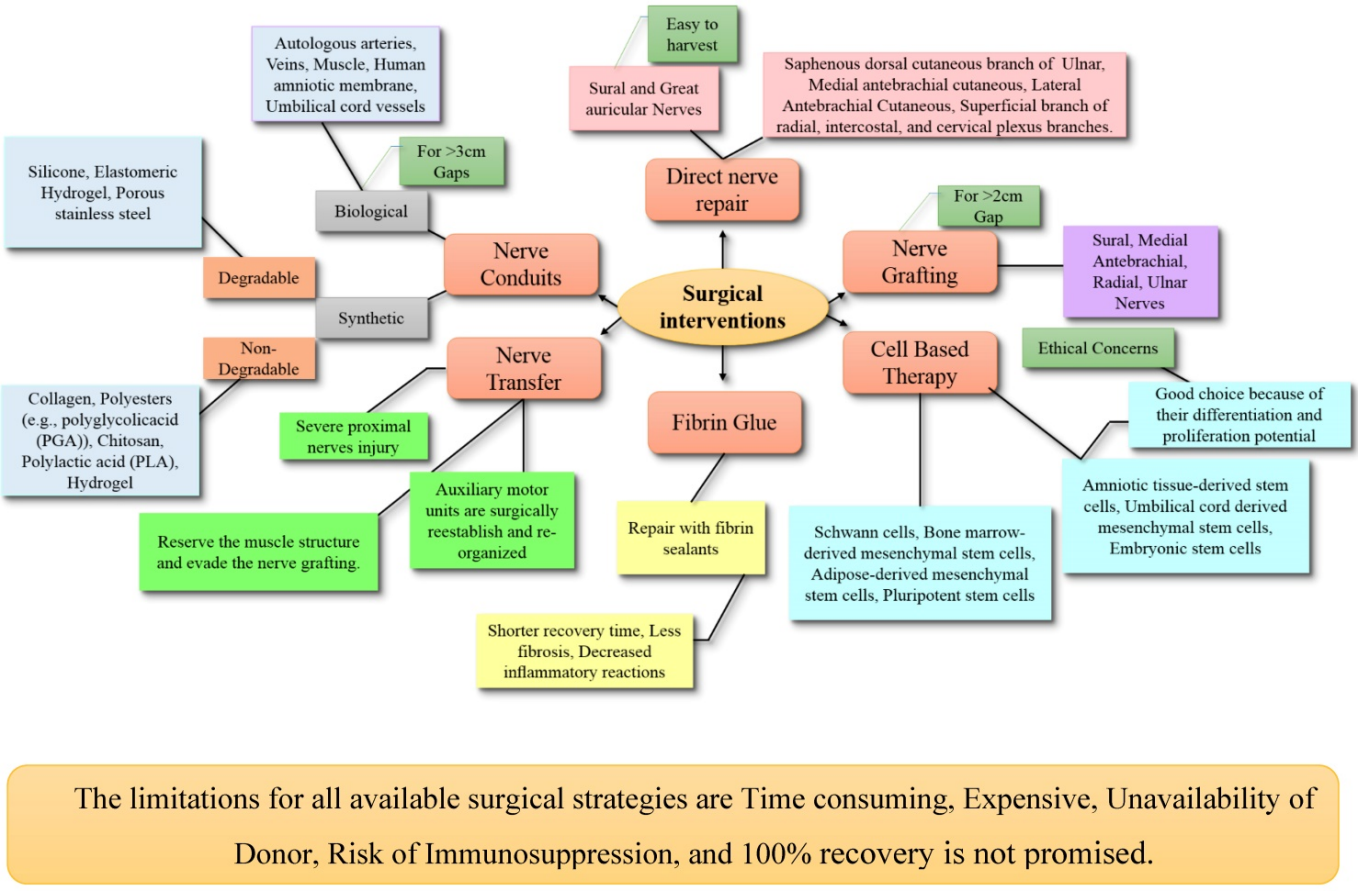
Surgical interventions for peripheral nerve repair

On the whole, it can be summarized that the ideal cells used for neural regeneration should have the following properties such as easy harvesting, no requirement of immunosuppression, able to integrate to the injury site and non-tumorigenic. The success of a cell-based therapy depends on the transplanted cell's ability to differentiate into Schwann-like cells, to release neurotrophic growth factors and to induce myelination of axons. Schwann cell cultures have mostly shown acceptable results in experimental studies, however, they are not good enough and search for an ideal cell is still ongoing. Most importantly, the neural stem cells manifest a plethora of significant effects that can ameliorate the nerve recovery process, so this should be taken into account for the future research on stem cell therapy. Even though the cell-based therapy is promising for the future, but it still lacks preclinical trials. The most important issues with this approach are; cell transplantation safety cell preparations are time-consuming and expensive. These delays might cause the aggravation of muscular atrophy rather than neuronal repair. All the available surgical interventions have been figured in figure 3.

### 2. Non-surgical therapeutic approaches for nerve recovery

Although surgical techniques for nerve repair are helpful, they are expensive and suboptimal. So, some of the non-surgical therapies are also under consideration such as medications and electrical nerve stimulation.

#### 2.1 Medications

**Figure 4:**
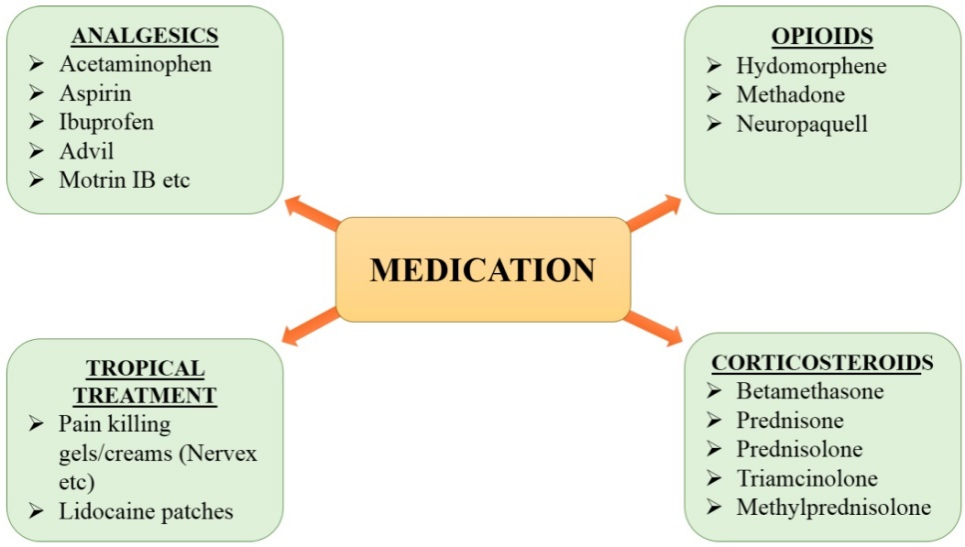
Available medications for nerve pain

Numerous categories of medication are available in the market to cure nerve pain but the best selection is tricky and it depends on the severity and cause of pain. The medications include analgesics, corticosteroids, gels and opioids (Fig 4). These are helpful in providing relief from pain and can also serve as the first-line option for treatment. Unfortunately, the available medications are not useful to treat the PNI because they can only calm the pain and cannot accelerate the nerve regeneration/functional recovery in severe cases.

#### 2.2 Electrical nerve stimulation

The repair of a nerve of any type leads to the state of short term or long term communication disturbance between the nerve and muscle. The target muscle stays denervated due to the deadly slow rate of regeneration that results in denervation-associated tissue atrophy. A direct method to attenuate the muscular atrophy is to excite the muscle electrically 125. Electrical stimulation plays an important role in the treatment of neuromuscular junctions' related diseases, 126. The electrical stimulation (ES) of NMJs is performed by applying electrical current directly to the skin surface and underlying muscle to persuade a muscle contraction that can help avoid the muscle atrophy during the period of reinnervation 127. To soothe muscle atrophy and recovery function of denervated muscle, stimuli should be applied several times a day at adequate intensity, pulse duration and frequency 128.

The timing to start electrical stimulation is quite questionable. The report suggests that the major improvement in twitching tension of crushed nerve was only noted when ES was applied during the middle period (day 12-21) after nerve crush, however, no effect was observed when applied at other time points. It clearly indicates that the stimulatory effects are obtainable only in a specific time window 128. On the functionally recovering NMJs, ES may exert an inhibitory effect when performed daily 129. There are many types and methods of electrical stimulation including percutaneous electrical nerve stimulation (PENS), Transcutaneous electrical nerve stimulation (TENS), Repetitive transcranial magnetic stimulation (rTMS) and Deep brain stimulation (DBS). In addition, instant high-frequency (100 Hz) electrical stimulation of the muscle exerts a remarkable increase in the expression of neurotrophic factors 130. Moreover, high-frequency electrical stimulation (200 Hz) executes a better effect on myelination than a low-frequency stimulation (20 Hz)^131^.

Although ES therapies are beneficial for nerve regeneration, they have also parade harmful effects. The TENS distort the morphology of axon with dark axoplasma, edema, and disorganized cytoarchitecture 132. In addition, a reduction in axon number has also been observed with thinner myelination but with the increased number of Schwann cell nuclei 133. The ES also reduces the muscle excitability, the integrity of neuromuscular junctions, neural cell adhesion molecules expression and muscle fiber cross-sectional area. The stimulation of a partly innervated muscle also casts undesirable effects for the remaining nerves because nerve connections to the muscle are shaped in an asynchronizing manner and stimulation at this time may compromise the functional re-innervation 128.

## Phytochemicals: an alternative source for available therapeutic approaches

Phytochemicals are plant-derived compounds, abundantly found in nature, have been traditionally used against a large number of diseases. They are getting attention because of having less or no menacing effects. They have been used for medicinal purposes from ancient time. Recently, Hussain et al., has well described the ameliorative role of different phytochemicals such as alkaloids, flavonoids and tannins against brain ailments 134-136. Moreover, they have also described the effective interventions of fatty acids such as lipids, cholesterol, and sphingolipids on a similar note 137,138. Many of them have also been used to treat many age-related health ailments like neurodegenerative diseases including PD, AD, and dementia 155. More than 80,000 of plant species are used in the world for the medicinal purpose 24,140, and about 80 % of the people depend on the plant-derived compounds for a major line of health care 156,157,158*.* Several compounds have been identified to accelerate the recovery process after PNI and they have been described below:

### 1. 4-Aminopyridine

4-Aminopyridine is a voltage-gated potassium channels blocker with reported ameliorating effects against different diseases of nervous system. The ability of 4-Aminopyridine (4-AP) is to promote durable recovery and remyelination following acute traumatic nerve injury indicates a valuable use of this compound to enhance nerve repair 144. It has been reported that in multiple sclerosis, regular 4-AP administration ameliorates the chronic walking disability 145. The ability of 4-AP to allow quick distinction between complete and incomplete nerve injuries. This drug can potentially be used to recognize lesions in which short-term cure with 4-AP to endorse strong recovery would be most likely favorable and also more quickly identify individuals for who timely surgical intervention is required to improve the chances of recovery 144.

### 2. Quercetin

Quercetin is a flavonoid which offers beneficial biological properties 146. Particularly, its anti-inflammatory and antioxidant activities are evident in various reports 147. Recently, neuroprotective and antioxidant effects of quercetin in a rat model of sciatic nerve crush injury have been explored. This work indicates the beneficial effects of quercetin in accelerating the nerve regeneration and shortening the recovery period in mild to moderate type of nerve injuries like crush injury 148.

### 3. Ursolic acid

Ursolic acid (UA) is a pentacyclic-triterpenoid which is abundantly found in herbs' leaves, flowers and fruits. It possesses antioxidant, antimicrobial, anti-inflammatory, hepatoprotective, immune-modulatory, anti-tumor, chemopreventive, cardioprotective, antihyperlipidemic and hypoglycemic properties 164. A study, conducted to explore the role of UA in nerve regeneration indicates that this agent has the potential to promote regeneration of the injured sciatic nerve in a mouse model 150.

### 4. Curcumin

Curcumin belongs to the polyphenol class of compounds extracted from plants of the genus Curcuma. It is beneficial in the management of oxidative stress, inflammatory situations, metabolic syndromes, arthritis, anxiety and hyperlipidemia 151. Studies have revealed that curcumin promotes nerve regeneration after a crush injury 152. The repair of peripheral nerve injury after complete amputation is difficult. Even with anastomosis, the rapid recovery of nerve function seems impossible. Recently it has been reported that curcumin has the potential to promote recovery after complete sciatic nerve amputation 153. Moreover, it also alleviates the neuropathic pain by reducing the activity of p300/CBP HAT which mediates the Cox-2 and BDNF expression 154. On top of that, curcumin possesses the capability to upregulate the expression of S100 protein and reduces the Schwann cells apoptosis which plays an important role in escalating the nerve regeneration 58,155.

### 5. 7, 8-dihydroxycoumarin

A plant-derived polyphenolic compound “7,8-dihydroxycoumarin” exhibits antimitotic, immune-modulating, antiviral, anticancer and cytotoxic effects 156. A study was conducted in a mouse model of sciatic nerve injury. The mouse was treated with an intraperitoneal injection of 7, 8-dihydroxycoumarin. The results indicate that 7, 8-dihydroxycoumarin promotes the repair of the injured nerve by up-regulating the expression of growth-associated protein-43 in the corresponding spinal cord segments of mice with sciatic nerve injury 157.

### 6. Red Propolis

Red propolis is famous for its anti-inflammatory and anti-oxidant activities. The hydro-alcoholic extract of red propolis was administered orally for a month after inducing axonotmesis of the sciatic nerve in a rat model. The behavioral and morphometric analysis had been done to measure the extent of recovery 158. The results of the study clearly indicated that the hydroalcoholic extract of red propolis has a capability to promote regenerative responses and accelerate the functional recovery after sciatic nerve crush. Thus, it can be a valuable and complementary therapy for healing nerve injuries 158.

### 7. Lycium babarum

Lycium babarum is a traditional medicinal herb and a food supplement which has been utilized by the Chinese for more than 2,000 years. It is a very rich source of betaine, phenolics, carotenoids, cerebroside, 2-O-β-d-glucopyranosyl-l-ascorbic acid (AA-2βG), β-sitosterol, flavonoids and vitamins (riboflavin, thiamine and ascorbic acid) 159. It also contains Lycium barbarum polysaccharides (LBPs) abundantly as an active compound that exhibits impressive antioxidant properties 160. Oral administration of these polysaccharides effectively promotes nerve regeneration. Thus Lycium barbarum polysaccharide would be a potential candidate to augment nerve regeneration following a nerve injury 161.

### 8. Tacrolimus

Tacrolimus is a macrolide immunosuppressant that is used to lower the risk of organ rejection after organ transplant 162. In a rat model with sciatic nerve transection, tacrolimus (4 mg/kg per day) for the period of 0, 2, 4 and 6 weeks significantly increased the average axon diameter, myelinated nerve fiber density and myelin sheath thickness 163. After sciatic nerve injury, intragastric administration of tacrolimus also significantly improved the sciatic functional index and gastrocnemius muscle net weight. The myelinated nerve fiber thickness in the nerve anastomosis and the sciatic nerve functions had a considerable negative association with the scar area, as detected by Spearman's rank correlation analysis. The available results indicate that tacrolimus remarkably improves peripheral nerve regeneration and accelerates the recovery of neurological functions by limiting scar formation 164.

### 9. Centella asiatica

*Centella asiatica* is an urban herb which is also known as Hydrocotyleasiatica L and has been used as a nerve tonic in Ayurvedic system of medicine for centuries 165. It has been reported that ethanolic extract of *Centella asiatica* (100μg mL-1) elicited a remarkable increase in neurite outgrowth in human SH‐SY5Y cells in the presence of nerve growth factor (NGF). Asiatic acid (AA) is a triterpenoid compound and found in ethanolic extract of *Centella asiatica,* has the potential to improve the neurite growth 166. Moreover, it also promotes rapid functional recovery and increases axonal regeneration. So, the components found in Centella ethanolic extract would be useful for speeding up the nerve repair 164.

### 10. Hericiumerinaceus-Mushroom

Hericiumerinaceus is a famous edible mushroom with tremendous medicinal properties and has been proved beneficial against Alzheimer's disease (AD), immune-regulatory disorders and cancer 167. It has been reported that the aqueous extract of Hericiumerinaceus fresh fruit bodies improves the axonal regeneration and re-innervation of the neuromuscular junctions (NMJs) in extensor digitorum longus muscle 168. It also improves the local axonal protein synthesis in the distal segments of the compressed nerves. In a rate model, daily oral administration of Hericiumerinaceus ameliorated the regeneration of injured peroneal nerve at the early stage of recovery 169. Moreover, oral administration of its aqueous extract promotes the peripheral nerve regeneration by modulating the signaling pathways i.e., Akt, MAPK, c-Jun, c-Fos and protein synthesis 170,171.

### 11. Lumbricus Extract

Lumbricus, commonly known as earthworm has a natural quality to regenerate its amputated body parts. This capability of earthworm has attracted the attention of scientists to explore its remedial properties 172. The extract has been used in China for centuries as a part of traditional Chinese medicines for promoting nerve functions by ameliorating the nerve conduction velocity 173. Moreover, the oral administration of such extract increases the regeneration and functional recovery after sciatic nerve compression injury 174.

### 12. Fermented soybeans (Natto)

Fermented soybeans (Natto) are enriched in menaquinone-7 which is effective in preventing osteoporosis 175. They share bioactive properties with tissue-type plasminogen activator which plays a crucial role in promoting nerve regeneration by clearing fibrin and inflammatory cytokines 176. The natto extensively enhanced injury-induced disorder of blood-nerve barrier and loss of matrix components such as fibronectin and laminin. Sciatic nerve crush injury increases the tumor necrosis factor-alpha (TNF-alpha) and causes apoptosis. The enhanced production of TNF-alpha and apoptosis were attenuated by natto treatment. It has been observed that the oral intake of natto accelerates the regeneration of peripheral nerve at a dose of 16mg/day. The speculated mechanism behind this effect is fibrin and decreased the production of TNF-alpha 177. On the basis of available data, although it can be summarized that natto is effective in promoting nerve regeneration but we were unable to find any latest report to affirm this idea.

### 13. Valproic acid (VPA)

Valproic acid is a famous anti-epileptic and mood-stabilizing drug 178,179. It has been demonstrated that it promotes the neurite outgrowth, activates the extracellular signal-regulated kinase pathway and increases bcl-2 and growth cone-associated protein 43 levels in the spinal cord 180. The rat sciatic nerve regeneration was enhanced through silicon tubes implanted with valproic acid 181. Moreover, a dose of 300mg/kg of valproic acid () 181 significantly enhances the sciatic nerve regeneration and motor functional recovery 182.

### 14. Radix Hedysari

Radix Hedysari is a herbal preparation and is frequently used in traditional Chinese medicines. It has been proved that aqueous extract of Radix Hedysari Prescription and modified Radix Hedysari Prescription help the regeneration of damaged peripheral nerves 183. Hedysari polysaccharides (HPS), a major ingredient, also increases the peripheral nerve regeneration after nerve injury in adult animals. An oral administration of 2 ml HPS liquid daily, 0.25 g/ml 184 improved the tibial function index (TFI), sciatic function index (SFI), peroneal nerve function index (PFI), conduction velocity and number of regenerating nerve fibers that signifies the possible clinical application of HPS for the treatment of peripheral nerve injury 185,186. Moreover, Hedysari extract can successfully promote the growth of lateral buds in the proximal nerve stump and considerably improves the magnification effect during peripheral nerve regeneration 187.

All these reported plants and plant-derived compounds can prompt a breakthrough to pin down the authentic products to accelerate the functional recovery. Thoroughgoing studies are required to put them into preclinical and clinical trials. Moreover, their dose-dependent studies and measuring toxicity level are highly concerned. Additionally, the particular molecular markers and pathways influenced by these compounds should also be addressed.

**Figure 5:**
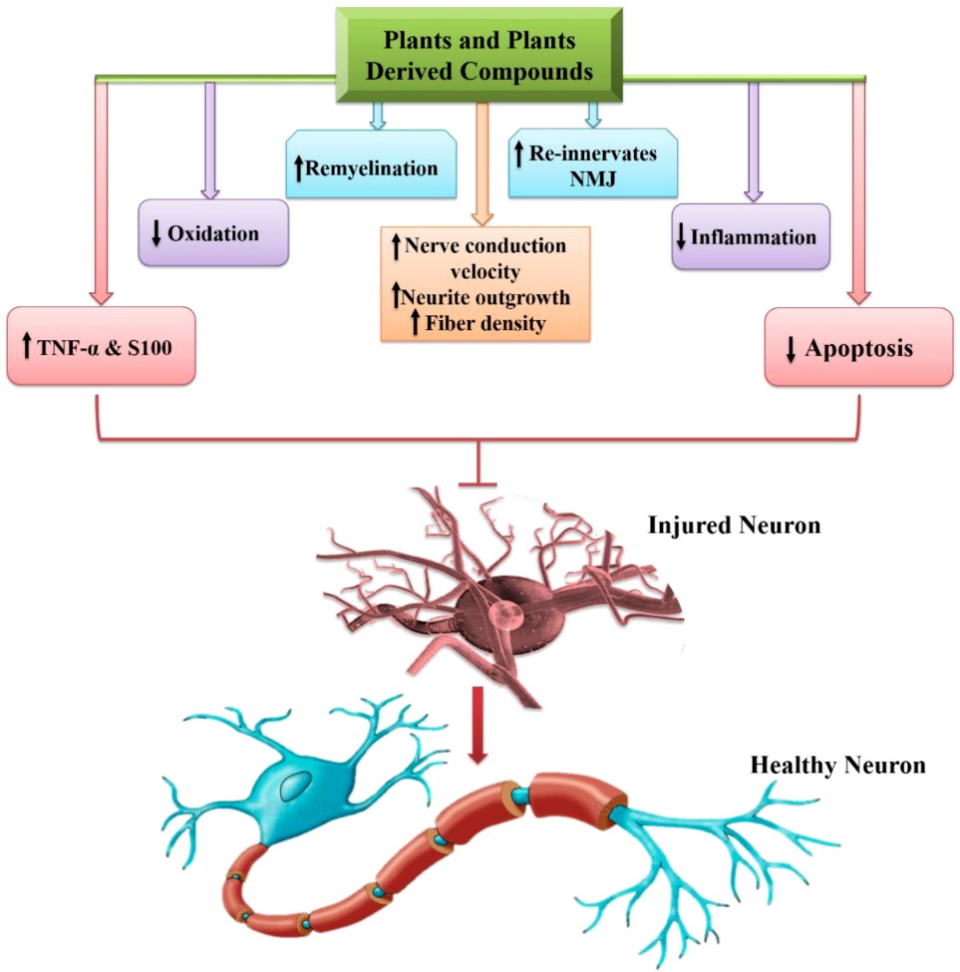
Phytochemicals and their role in peripheral nerve ingury

Lastly, some of the pivotal roles of phytochemicals regarding PNIs are presented in figure 5.

In table 4, the reported phytochemicals having nerve regeneration/functional recovery promoting ability following peripheral nerve injury have been summarized.

## Conclusion and future prospects

Peripheral nerve injury, one of the prominent health-related issues, exhibits a broad range of signs and symptoms depending on the intensity of nerve damage. Although a vast set of data regarding the pathological mechanisms of PNI and its regeneration is available, reliable treatment ensuring complete and accurate functional recovery is yet scanty. The recovery process is deadly slow and complete functional regain is still a dream even though various therapeutic strategies are in practice. In this review, we have tried to illustrate the advantages and limitations of available treatments against PNIs. Presently, both surgical and non-surgical strategies are valuable in regard to the PNIs' treatment. Unfortunately, surgical methods are quite expensive and their use has been limited due to various drawbacks such as immunosuppression, chromosomal aberrations, tumorigenicity and many more. On top of that, non-surgical interventions exhibit several advantages such as ease of use, minimizing tissue trauma and maintained the architecture of a nerve. Therefore, non-surgical interventions including medications and electrical nerve stimulation for promoting functional retrieval following PNIs are prioritized. Unluckily, the available medications are only symptomatic such as analgesics and corticosteroids and thus, remain suboptimal for accelerating the functional outcomes. Fascinatingly, plants and plants derived compounds have given a ray of beacon to the scientists due to their pharmacological properties, including anti-inflammatory, anti-oxidative, anti-microbial, neuroprotective and analgesic. Several of such compounds have been investigated such as curcumin, quercetin, radix hedysari, ursolic acid, *Centella asiatica* and red propolis which strengthen the idea of using such compounds. In spite of extensive research, still, there is a need for more work to advance the field of therapeutics for promoting peripheral nerve regeneration. As discussed in this review, many momentous advances in nerve repair and regeneration have been achieved but clinical trials are needed. A lot of regeneration-promoting factors have been recognized which are aspirational for the evaluation of these factors at the clinical level to promote the escalated nerve recovery. The natural compounds based on therapeutic strategies can prove an alternative to surgical interventions. In the future, these compounds are ought to be advanced at the clinical level after confirming their validity. It will be a great step to support the impoverished community of developing countries who cannot afford the expensive treatments.

## Figures and Tables

**Table 1:** Seddon's Classification - General Features

Neuropraxia	Axonotmesis	Neurotmesis
There is an occurrence of paralysis but the peripheral degradation is absent 11. In this kind of damage, the action potential spreading capability of the nerve becomes partially or completely lost, but the essential axonal continuation remains entirely preserved. This situation is connected with the demyelination of the nerve fibers segmentally 12. The motor neuronal fibers are most susceptible to this injury and they lose their functioning capability at first and re-gain at last. The example of neuropraxia is “Saturday night palsy” in which pressure occurs on nerve while sleeping. This condition generally improves in 12 weeks with no intervention 30.	The second grade of injury is axonotmesis in which the nerve fibers get severely damaged and leads to the intact peripheral deterioration 14,15. In this type of injury, the layers of connective tissue and the closely linked structures with nerve fibers remain conserved to some point so that the internal structures remain conserved suitably 33. Here, the entire Wallerian degeneration and axonal re-growth take place and the retrieval is good and spontaneous but not as worthy as neuropraxia. In this, the surgical intervention is generally not needed 17.	The 3^rd^ grade of nerve injury is neurotmesis which results in the injury of neural connective tissue constituents and effects perineurium epineurium, and/or endoneurium. The nerve fiber is entirely divided into two ends and leads towards whole paralysis 18. The Wallerian degeneration and axonal re-growth is a distinct property of this injury. In this, the regeneration process is restricted by intraneural damaging, axonal misdirection, and loss of blood-brain barrier. The injuries leading to the damage of epineurium and perineurium mean that surgical intervention becomes inevitable for recovery 17,19.

**Table 2:** Seddon and Sunderland classification of nerve injuries

Seddon classification	Neuropraxia	Axonotmesis	Axonotmesis	Axonotmesis	Neurotmesis	
Sunderland classification	Grade 1	Grade 2	Grade 3	Grade 4	Grade 5	Grade 6 (According to MacKinnon)
Causes	Local ischemia, traction, mild crush, compression	Nerve crush	Nerve crush	Nerve crush	Nerve laceration and transection	Stab or gunshot wounds, closed traction damage
Recovery	Complete - hours up to a few weeks	Complete - weeks to months	Incomplete and variable - months	Incomplete and variable - depending on injury andtreatment - months to years	Incomplete - months to years	Incomplete - months to years
Pathophysiology	Connective tissues and axons in continuity, nerve conduction block	Division of axons but all layers of connective tissues remain intact	Myelin sheath & endoneurial layer are disconnected.	Axon with myelin sheath, endoneurium and perineurium disconnected	Axon with myelin sheath, endoneurium, perineurium, and epineurium disconnected	Mixed injuries, all grades involved
Surgical Intercessions	Typically not	Typically not	Typically not	Typically required; procedure depends upon findings	Required; Early nerve healing or reconstruction	Surgical investigation & intraoperative electro-diagnostic techniques; nerve re-construction ornerve transferring

**Table 3:** Factors associated with peripheral nerve regeneration

Regeneration associated factors	Role in nerve regeneration	References
Activating Transcription Factor-3 (ATF-3)	The overexpression of ATF-3 promotes neurite outgrowth.	48
SRY-box containing gene 11 (Sox11)	It promotes peripheral nerve regeneration by regulating the factors essential for neuronal survival and neurite outgrowth.	49
c-Jun	It appears to increase the expression of other regeneration associated genes (RAGs) and in that way may promote a growth state. Moreover, it is important for the activation of the nerve repairing program and its absence leads to the inactivation of significant several cell surface proteins and trophic factors which sustain the survival and axonal growth.	40
Small proline-repeat protein 1A (SPRR1A)	It is undetectable in the uninjured neuron but its expression increase by 60-folds after damage to the peripheral axon. It is a substantial contributor to the effective nerve regeneration, hence its reduction restricts the axonal outgrowth.	50,51
Growth-associated protein-43 (GAP-43)	It is a marker for neural regeneration and outgrowth. Its overexpression results in the spontaneous new synapses formation and increased sprouting after nerve injury	52,53
Agrin protein	It is a nerve derivative protein, secreted by motor neurons into the synaptic cleft. It forms the AChRs clusters on the emergent skeletal muscle fiber that may assist as a target for the innervating motor neurons. It acts through the muscle-specific tyrosine kinase (MuSK) initiating the signaling pathway leading to the rapsyn-reliant AChR clustering. It also promotes the development of filopodia on neurites by increasing the number and stability of these filopodia.	54-57
S100 protein	It promotes the proliferation of Schwann cells significant in neural regeneration. S100B protein expresses in Schwann cells upon the acute peripheral injury to the nerve which is released by the Schwann cells in injured nerves stimulates RAGE in infiltrating macrophages and in the activated Schwann cells. Moreover, the S100B activated RAGE endorses the migration of Schwann cells by activation of p38, NF-κB, CREB, and MAPK.	58-60
CCAAT/enhancer binding protein delta (C/EBPd) and C/EBP-like transcription factor genes	They are found to be up-regulated abruptly after nerve injury. They are involved in the lipid metabolism regulation and activation of macrophage.	61,62
Nerve growth factor (NGF), Fibroblast growth factor (FGF), Ciliary neurotrophic factor (CNTF), Interleukin-like growth factor (IGF), Vascular endothelial growth factor (VEGF), and Brain-derived neurotrophic factor (BDNF)	The expression of a large number of neurotropic factors/growth factors increased in Schwann cells of the distal stump after nerve injury. These neurotropic factors are released by the Bands of Bungner's that support the neuronal survival and promote remyelination. Moreover, they also monitor the sprouting axon into the endoneurial column.	41-44
Surface cell adhesion molecules (CAMs), including L2/HNK-1, Ng-CAM/L1, N-CAM, and N-cadherin	The production of these factors enhanced by the surviving Schwann cells to promote nerve regeneration/ remyelination.	86
Tenascin, heparan sulphate proteoglycans (HSP), fibronectin (FN), and laminin (LN)	These are the extracellular matrix proteins found in the basement membrane of Schwann cells promoting remyelination.	86

**Table 4:** Remedial approaches to promote nerve recovery

Remedies	Activity	References
4-Aminopyridine	Promotes remyelination	144
Quercetin	Anti-inflammatory, antioxidant & neuroprotective.	148
Ursolic acid	Antioxidant, antimicrobial, anti-inflammatory, hepatoprotective, immune-modulatory, anti-tumor, chemopreventive, cardioprotective, anti-hyperlipidemic and hypoglycemic.	165
Curcumin	Manages metabolic syndromes, arthritis, anxiety, oxidative stress, inflammatory situations. Enhances the expression of S100.	58,151
7,8-dihydroxycoumarin	Antimitotic, immune-modulating, antiviral, anticancer and cytotoxic effects.	156
Red propolis	Anti-inflammatory and anti-oxidant activities.	158
Lycium babarum	Anti-oxidant.	160
Tacrolimus	Increased average axon diameter, myelinated nerve fiber density and myelin sheath thickness.	163
*Centella asiatica*	Improve neurite outgrowth & axonal regeneration.	165
Hericiumerinaceus Mushroom	Re-innervation of the neuromuscular junction.	168
Lumbricus Extract	Improves nerve regeneration, functional recovery, and nerve conduction velocity	173,174
Fermented soybean	Promote nerve regeneration by increasing TNF-α and decreasing apoptosis	177
Valproic acid (VPA)	Anti-epileptic & mood stabilizing agent.	182
Radix Hedysari	Neuronal regeneration.	185,186

## Abbreviations

CNS: Central Nervous system
PNS: Peripheral Nervous system
PPNIs: Preoperative peripheral Nervous system
WD: Wallerian degeneration
BMSCs: Bone marrow-derived mesenchymal stem cells
ADSCs: Adipose-derived mesenchymal stem cells
NSCs: Neural stem cells
ESCs: Embryonic stem cell
ATDSCs: Amniotic tissue-derived stem cells
UC-MSCs: Umbilical cord derived mesenchymal stem cells
ADSCs: Adipose stem cells
SKP-SCs: Skin-derived precursor stem cells
HFSCs: Hair follicle stem cells
ES: Electrical stimulation
PENS: Percutaneous electrical nerve stimulation
TENS: Transcutaneous electrical nerve stimulation
rTMS: Repetitive transcranial magnetic stimulation
DBS: Deep brain stimulation
AD: Alzheimer's disease
PD: Parkinson's disease
LBPs: Lycium barbarum polysaccharides
HPS: Hedysari polysaccharides
TFI: Tibial function index
SFI: Sciatic function index
PFI: Peroneal nerve function index
MRI: Magnetic resonance imaging
PLA: Polymers are polylactic acid
PGA: Polyglycolic acid
PCL: Poly-caprolactone
PLCL: Poly-lactidecaprolactone
ATF-3: Activating Transcription Factor-3
FGF: Fibroblast growth factor
NGF: Nerve growth factor
IGF: Interleukin-like growth factor
CNTF: Ciliary neurotrophic factor
BDNF: Brain-derived neurotrophic factor
VEGF: Vascular endothelial growth factor
